# Charge Effects: Influence of Surface Charge on Protein Corona Adsorption Behavior on Liposomal Formulations

**DOI:** 10.3390/pharmaceutics18010076

**Published:** 2026-01-07

**Authors:** Qian Chen, Yeqi Huang, Chuanbin Wu, Xin Pan, Changjiang Yu, Jiu Wang, Wenhao Wang, Zhengwei Huang

**Affiliations:** 1 Department of Pharmacy, College of Pharmacy, Jinan University, Guangzhou 511436, China; 2 State Key Laboratory of Bioactive Molecules and Druggability Assessment, Guangdong Basic Research Center of Excellence for Natural Bioactive Molecules and Discovery of Innovative Drugs, College of Pharmacy, Jinan University, Guangzhou 511436, China; 3 School of Pharmaceutical Sciences, Sun Yat-sen University, Guangzhou 510006, China; 4 Key Laboratory of Natural Polymer Function Material of Haikou City, College of Chemistry and Chemical Engineering, Hainan Normal University, No. 99 Longkunnan Road, Haikou 571158, China; 5 Guangdong Provincial Key Laboratory of Pharmaceutical Preparations Research and Evaluation, Higher Education Mega Center, Guangdong Pharmaceutical University, No. 280, East Waihuan Road, Guangzhou 510006, China; 6 Key Laboratory of Biological Molecular Medicine Research (Guangxi Medical University), Education Department of Guangxi Zhuang Autonomous Region, Nanning 530021, China

**Keywords:** liposomes, protein corona, DOTAP, magnesium chloride, interaction models

## Abstract

**Background**: Liposomes have been successfully used in clinics as an excellent drug delivery system. However, once they enter the body, they adsorb surrounding proteins and form a protein corona, which affects how liposomes behave in vivo. Therefore, controlling the formation of the protein corona is crucial for achieving effective treatment outcomes. Among the many variables affecting liposome protein corona formation, the composition of the liposomes themselves and the surrounding ionic environment are two particularly critical factors. **Methods**: In this context, this study selected bovine serum albumin as a model protein to investigate the influence and mechanism of physiologically relevant inorganic ions (magnesium chloride) and varying proportions of cationic lipid components (1,2-dioleoyl-3-trimethylammonium-propane (DOTAP)) on protein adsorption behavior of liposomes. We evaluated characterization parameters, including particle size and zeta potential, and employed various spectroscopic techniques to elucidate the changes during the interaction between bovine serum albumin and liposomes. **Results**: The zeta potential results showed that liposomes without DOTAP exhibited a significantly negative potential (−45.20 ± 0.24 mV), while the zeta potential became increasingly positive with higher DOTAP proportions (+19.64 ± 0.39 mV and +51.03 ± 1.74 mV). Correspondingly, the amount of protein adsorption also increased with the rising DOTAP content. Furthermore, fluorescence spectroscopy indicated that the addition of either DOTAP or magnesium ions led to a decrease in both the *K*_sv_ and *K*_a_ parameters. **Conclusions**: Specific hypothetical models were advanced subsequently; per the varying proportion of DOTAP, we proposed an insertion or surface adsorption model, and further examined the influence of magnesium chloride on the interactions between the liposomes and proteins. We believe this study will provide a new research paradigm for the design and application of liposomes, laying a foundation for further in vivo investigations.

## 1. Introduction

Liposomes have emerged as extensively studied and versatile nanocarriers for drug delivery, owing to their excellent biocompatibility, biodegradability, and capacity to encapsulate a wide range of therapeutic agents. Their applications encompass multiple therapeutic areas, including gene therapy and vaccine development via the delivery of nucleic acids such as mRNA or siRNA [1], antibacterial treatment through the encapsulation of antibiotics [2], and cancer therapy by the delivery of chemotherapeutic drugs [3].

Nevertheless, only a limited number of liposomal formulations, like doxorubicin liposomes [4] and amphotericin B liposomes [5], have achieved formal clinical approval to date. This reality underscores the considerable challenges that persist in bridging the gap between foundational research and successful clinical translation and commercialization. The complex in vivo fate of liposomes and the unpredictable nature of their behavior within the human body represent major obstacles to their broader clinical adoption. Upon entering a biological environment, a multitude of factors, including blood components, immune responses, physiological barriers, and individual patient differences, can significantly alter their journey and efficacy. They rapidly adsorb biomolecules such as proteins, leading to the formation of a protein corona [6]. For example, while studying the anticancer effects of PEGylated liposomal doxorubicin (PLD), the first FDA-approved liposome for cancer therapy, in obese mouse models, Xu et al. observed that apolipoprotein E, highly expressed in the plasma of obese individuals, actively shapes the composition of the protein corona on the liposomal surface, thereby influencing its biological fate. Results showed that this “obesity-related protein corona” significantly reduced macrophage clearance of the liposomes, extending their circulation time. It also promoted targeted accumulation of the liposomes in tumor sites and enhanced their uptake by cancer cells. This specific protein corona, shaped by the obese microenvironment, could significantly influence macrophage-mediated clearance, circulation half-life, and tumor accumulation of the liposomes [7]. Similar studies indicated that the rapidly formed protein corona in vivo also influenced processes such as hemolytic reactions, platelet activation, and nanoparticle uptake [8], ultimately affecting the therapeutic efficacy of the drug. It is well established that egg lecithin liposomes represent a classic type of nanocarrier with excellent biocompatibility, holding significant value both as a model system and for translational research in drug delivery. The core phospholipid component, egg lecithin, closely mimics the composition of cell membranes, providing the liposomes with favorable biocompatibility and low immunogenicity. This has established them as foundational materials in early-stage liposomal research. However, like all intravenously administered nanocarriers, the surface properties, in vivo fate, and biological behavior of egg lecithin liposomes are also regulated by the protein corona upon entering the systemic circulation [9,10]. Therefore, while advancing the clinical application of liposomes, in-depth research into their interactions with biological systems is crucial for developing safer and more efficient nanomedicines.

It is widely reported that the formation of protein corona on liposomes can be tuned by various factors. Among them, the lipid composition of the liposomes and the ionic environment of the biological fluid are two particularly critical determinants. The chemical composition of liposomes directly determines their initial interaction patterns with blood proteins [11]. The phospholipid type serves as a key factor regulating liposomal surface charge, with cationic lipids being extensively studied for their role in promoting protein adsorption through electrostatic interactions, ultimately leading to protein corona formation [12,13]. Among various cationic lipids, 1,2-dioleoyl-3-trimethylammonium-propane (DOTAP) stands out as one of the most extensively studied and widely used in both research and applications. DOTAP features a strongly positively charged trimethylammonium group. It enables the effective adsorption of negatively charged biomolecules in physiological environments, facilitating the formation of stable liposomal nanocomplexes. Meanwhile, its two unsaturated oleoyl chains confer dynamic fluidity and structural flexibility to the lipid bilayer [14]. Studies have shown that DOTAP may play a key role in targeted cell delivery by altering the surface charge of lipid nanoparticles (LNPs) [15]. Moreover, DOTAP-based LNPs have been extensively studied and widely used in both preclinical and clinical settings [16,17,18]. For example, EndoTAG-1 (ET), a liposome that uses DOTAP as its cationic lipid to encapsulate paclitaxel (Taxol), has advanced to Phase III clinical trials. These trials are evaluating its ability to target tumor endothelial cells in solid tumors [19]. Additionally, a cationic LNP gene delivery system composed of DOTAP and cholesterol is being tested in numerous clinical studies with the potential to enhance the delivery of nucleic acid-based vaccines and therapeutics [20]. In summary, DOTAP is widely studied and highly regarded for its applications, making it an ideal model and a central research subject in the field of cationic lipid studies.

Simultaneously, when these cationic liposomes enter a physiological environment, their surface charge is not static. It quickly changes in response to the surrounding ions, which influence both the distribution and magnitude of the charge. This dynamic interaction significantly affects how proteins adsorb onto the nanoparticle surface [21,22,23]. Currently, in scientific studies, anions such as chloride (Cl^−^) are primarily viewed as background components that maintain electroneutrality [24]. In contrast, cations tend to play more active and critical roles in a wide range of biological processes, making them the central focus of research. Research has shown that divalent cations are likely to play a far more complex and critical role in protein corona formation than monovalent ions. This is attributed to their higher valence, which results in greater charge density, and their smaller ionic radii, which enhance their coordination capacity [25]. Notably, among various divalent cations, the magnesium ion (Mg^2+^) holds a unique position, making it a highly valuable research model. Mg^2+^ is the second most abundant intracellular cation in the human body after potassium and is present at considerable levels in plasma, with approximately 55–60% in the physiologically active free-ion form [26]. This implies that any intravenously administered nanomedicine is immediately and continuously exposed to an environment containing Mg^2+^. Furthermore, Mg^2+^ serves as an essential cofactor for over 300 enzymatic reactions and participates broadly in key biochemical processes, including energy metabolism, protein synthesis, and the regulation of blood glucose and blood pressure [26,27]. This inherent biocompatibility and biological relevance suggest both the ubiquity and importance of its interactions with biomacromolecules. Consequently, selecting Mg^2+^, which is highly abundant in physiological environments, as a research model carries direct practical significance. Given that previous studies have predominantly investigated the effects of either liposome composition [28,29] or ionic environment [30] in isolation, in this study, we integrated both factors within a unified framework to systematically examine their respective roles in protein corona formation. In addition, bovine serum albumin (BSA), which shares a high degree of similarity with human serum albumin (HSA) (76% sequence identity) [31,32], has been widely adopted as a substitute model for HSA to investigate protein corona characteristics.

Based on the above rationale, our study used BSA as a model protein, magnesium chloride (MgCl_2_) at physiological concentration (Mg^2+^ at 20 μg/mL) [26] as the ionic medium, and DOTAP as the liposome surface charge regulator. We systematically explored how varying the DOTAP ratio in liposomes altered their BSA adsorption behavior, culminating in the proposal of two distinct interaction hypothetical models. The study further examined inorganic ions in liposome–protein interactions. Subsequent spectroscopic measurements, including fluorescence spectroscopy, ultraviolet-visible (UV-Vis) spectroscopy, and circular dichroism (CD), revealed and validated the proposed protein corona formation patterns on liposomes through detected changes in protein characteristics. This study is expected to provide systematic insights into the in vivo fate of liposomes and the formation mechanisms of the protein corona.

## 2. Materials and Methods

### 2.1. Materials

All chemicals used in this study were of reagent or analytical grade and were sourced from reputable commercial suppliers. All aqueous solutions were prepared using products from Yingmao (Guangzhou, China). Egg yolk lecithin (CAS: 93685-90-6) was purchased from Solarbio (Beijing, China). Cholesterol (CAS: 57-88-5), MgCl_2_ (CAS: 7786-30-3), DOTAP (CAS: 144189-73-1), BSA (CAS: 9048-46-8), and Coumarin 6 (C6) (CAS: 38215-36-0) were supplied by Shanghai Macklin Biochemical Technology Co., Ltd. (Shanghai, China). The RAW 264.7 cell line was obtained from Procell (Wuhan, China), and FBS was sourced from Thermo Fisher Scientific (Shanghai, China).

### 2.2. Preparation and Characterization of Liposomes

Liposomes were prepared using the thin-film ultrasonic dispersion method. Precisely weighed yolk phospholipids and cholesterol at a mass ratio of 5:1, along with varying proportions of DOTAP, were dissolved in 5 mL of dichloromethane. Remove the organic solvent by rotary evaporation for 5 min using a rotary evaporator (BPZ-6210-2, Changshenglong Instrument Co., Ltd., Guangzhou, China) to ultimately form a uniform lipid film at the bottom of the round-bottom flask. Subsequently, dissolve the film with 5 mL of ultrapure water and hydrate at 37 °C for 90 min. The resulting crude liposome suspension was processed in an ice bath using an ultrasonic cell disruptor (BILON-650Y, Mingli Instrument Equipment Co., Ltd., Guangzhou, China) at 450 W for 6 min, with the pulse turned off for 1.5 s after 1.5 s sonication to prevent heat build-up, thereby obtaining the final liposome suspension. The particle size, PDI, and zeta potential of the prepared liposomes were determined using a Malvern Mastersizer 2000^®^ (Malvern Instruments Ltd., Malvern, UK), while particle concentration and size distribution were analyzed with a NanoSight NS300 nanoparticle tracking analysis system (Malvern Instruments Ltd., Malvern, UK). During particle size, PDI, and zeta potential measurements, the instrument autonomously determines the number of scans based on the state and properties of the solution. The final reported results were derived from the fitting of data obtained through these multiple scans.

### 2.3. Preparation of Liposomes Incubated with BSA

Liposomes, BSA, and MgCl_2_ solution (2 mL for each) were mixed in equal volumes to achieve final concentrations of 1.2 mg/mL for liposomes, 0.8 mg/mL for BSA, and 20 μg/mL for MgCl_2_. Subsequently, appropriate amounts of BSA and MgCl_2_ were dissolved in 6 mL of ultrapure water to serve as the control group (the concentrations of both were the same as those in the experimental group). The mixture was incubated at 37 °C with continuous shaking at 100 rpm for 2 h. For the control group, BSA was dissolved directly in ultrapure water as the solvent for parallel experiments. To confirm the formation of a protein corona on the liposomes, the particle size and zeta potential were measured using a Malvern Mastersizer 2000^®^ after incubating the liposomes with varying concentrations of protein.

### 2.4. Protein Adsorption Analyzed by BCA Assay

Following incubation, the mixture was centrifuged using ultrafiltration tubes with a molecular weight cutoff of 100 kDa. Unbound proteins were separated by ultrafiltration centrifugation at 15,000 rpm for 30 min at a temperature of 4 °C. The control group was also subjected to the same centrifugation process, and the measured amount of BSA was used as the “total protein amount” for calculation. The collected unbound BSA was subjected to protein concentration determination using the BCA assay, and the amount of protein adsorption was calculated based on the fluorescence intensity at 562 nm. All measurements were performed in triplicate.

### 2.5. Fluorescence Spectra

In the fluorescence quenching assay, BSA was incubated with liposomes at different concentrations under the conditions described in Section 3.3. The fluorescence intensity of the BSA solution without liposomes and the blank liposome solution served as the baseline reference to correct for scattering and inner filter effects. After incubation, 2 mL of the solution was transferred to a quartz cuvette, and liposome solutions with concentrations ranging from 0 to 2.33 mg/mL were measured using a Fluoromax-4 spectrofluorometer (HORIBA, Kyoto, Japan). Fluorescence spectra were recorded with an excitation wavelength of 280 nm, 4 nm bandwidth, and emission scanning range of 300–460 nm. The obtained data were analyzed using the Stern–Volmer equation. In Equation (1), *F*_0_ and *F* represent the fluorescence intensity of BSA without and with liposomes, respectively. *K*_q_ is the bimolecular quenching rate constant, *τ*_0_ is the average fluorescence lifetime of the biomolecule, and [*Q*] is the concentration of liposomes incubated with BSA.

### 2.6. UV-Vis Spectra

After incubation of BSA with liposomes, the 2 mL aliquot of the solution was transferred to a 1.0 cm quartz cuvette. Ultrapure water was used as the reference to calibrate the baseline at room temperature with a scanning step size of 1 nm. The UV-Vis spectrum from 200 nm to 800 nm was recorded using a UV-Vis spectrophotometer (UV-2600, Shimadzu Corporation, Tokyo, Japan).

### 2.7. CD Spectra

The 400 μL aliquot of the BSA-liposome incubation solution was transferred to a quartz sample cell for scanning using a Chirascan circular dichroism spectropolarimeter (Applied Photophysics Ltd., Leatherhead, Surrey, UK) at room temperature. The scanning parameters were set as follows: bandwidth 1 nm, spectral range 190–260 nm, with continuous nitrogen gas purging during operation. Each sample underwent three consecutive scans, and baseline correction was performed using the CD spectrum of ultrapure water.

### 2.8. Cellular Uptake

RAW 264.7 cells were cultured in high-glucose DMEM supplemented with 10% FBS and 1% penicillin-streptomycin at 37 °C in a 5% CO_2_ incubator. Cells were seeded in 6-well plates for FCM analysis and incubated for 24 h. Subsequently, C6-containing liposomes were prepared via the thin-film dispersion method. Liposomal formulations with or without FBS were obtained following the procedure described in Section 3.3. The prepared plates were taken out, and the complete medium was discarded. Then, 1 mL of the five-fold diluted liposomal formulation was added, followed by incubation for 4 h. After incubation, the drug solution was discarded, and the cells were gently washed twice with 1 mL of PBS buffer. Subsequently, the cells were detached by pipetting with 1 mL of PBS buffer, and the resulting suspension was transferred to a centrifuge tube and centrifuged at 1200 rpm for 3 min. The cell pellets from each group were then resuspended in 1 mL of PBS buffer. Finally, the suspensions were transferred to pre-chilled flow cytometry tubes maintained on ice. The cellular uptake of the liposomes was quantified by measuring the fluorescence intensity of C6 using the 488 nm excitation channel.

## 3. Results and Discussion

### 3.1. Characterization of Particle Size, Zeta Potential, and Adsorption

**Figure 1:**
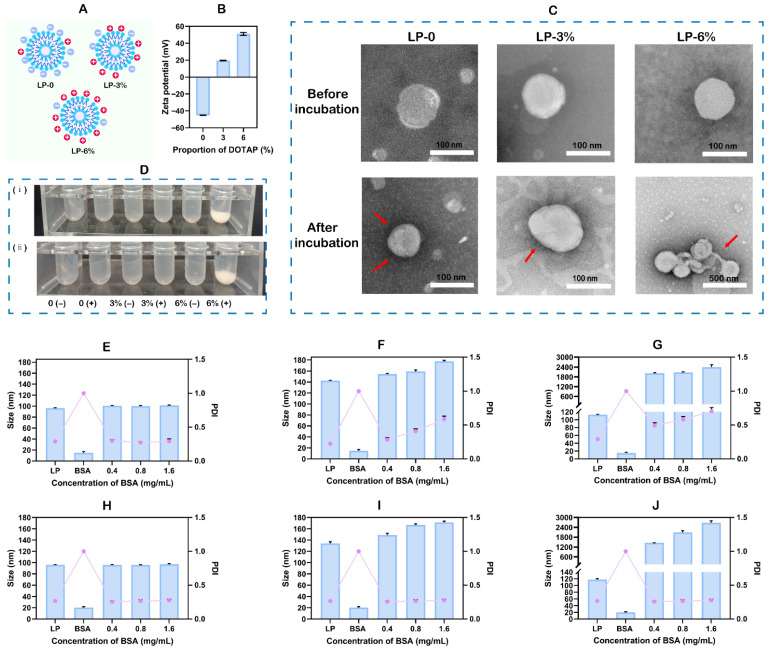
(**A**) Schematic diagrams of liposomes with varying DOTAP ratios. (**B**) Zeta potential of the liposomes. (**C**) The TEM images of liposomes before and after incubation with BSA. The red arrows indicate the adsorbed BSA. (**D**) The images of liposomes with (+) or without (−) BSA in tubes under conditions (**i**) without MgCl_2_ and (**ii**) with MgCl_2_. The particle size (columns) and PDI (line) of (**E**,**H**) LP-0, (**F**,**I**) LP-3%, and (**G**,**J**) LP-6% after incubation with BSA at concentrations of 0.4, 0.8, and 1.6 mg/mL. (**E**–**G**): without MgCl_2_, (**H**–**J**): with MgCl_2_. Data are presented as mean ± SD (*n* = 3).

**Figure 2:**
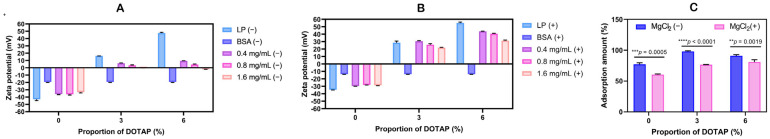
The zeta potential of liposomes incubated with BSA at different concentrations (0.4, 0.8, 1.6 mg/mL) under (**A**) without MgCl_2_ (−) and (**B**) with MgCl_2_ (+) conditions. (**C**) Percentage of BSA adsorbed by liposomes. Data are presented as mean ± SD (*n* = 3).

Firstly, liposomes with DOTAP ratios of 0%, 3%, and 6% were prepared using the thin-film dispersion method, and the zeta potentials of the three formulations were determined. The liposomes without DOTAP (LP-0) exhibited a zeta potential of −45.20 ± 0.24 mV, those with 3% DOTAP (LP-3%) showed +19.64 ± 0.39 mV, and those with 6% DOTAP (LP-6%) registered +51.03 ± 1.74 mV (Figure 1B). Subsequently, based on preliminary experiments, BSA at a concentration of 0.8 mg/mL was incubated with the liposomes to trigger protein corona formation. Transmission electron microscopy (TEM) images revealed a distinct dark layer of consistent thickness around the liposomes (Figure 1C), confirming the successful formation of the protein corona. Furthermore, it was also observed that as BSA concentration increased, the particle size of the LP-0 group remained largely unchanged. Interestingly, the LP-3% and LP-6% groups exhibited concentration-dependent increases in particle size (Figure 1E–J). Moreover, we measured the zeta potential of liposomes incubated with varying protein concentrations (Figure 2A,B). It is hypothesized that when proteins are adsorbed, the surface properties of the protein corona-coated nanocarriers will shift toward those of the adsorbed proteins. The results showed that as the protein concentration increased, the zeta potential of the liposomes gradually shifted, approaching that of the protein itself, indicating the formation of a protein corona. Moreover, the change in zeta potential for LP-0 after BSA adsorption was significantly smaller than that observed for LP-3% and LP-6%. Furthermore, the adsorption amount of LP-0 was significantly lower than that of LP-3% and LP-6%, as shown in Figure 2C. Our results strongly align well with the study by Jang et al., which demonstrated that negatively charged, highly PEGylated surfaces favor adsorption of lipids and hydrophilic metabolites, while positively charged, minimally PEGylated surfaces show a preference for protein adsorption [33]. Hence, our findings provide experimental confirmation that surface charge plays a decisive role in shaping the protein corona composition on nanocarriers. These collective phenomena suggested that different protein corona formation models may exist among the three liposome types.

### 3.2. The Insertion Model and the Surface Adsorption Model

Based on the above findings, we proposed two distinct hypothetical models corresponding to the different DOTAP ratios in the liposomes: the insertion model and the surface adsorption model:

(i) Insertion model. When the liposome contains no DOTAP, its surface is negatively charged (Figure 1A). The presence of negatively charged BSA (isoelectric point ≈ 4.8) [34] in the system creates electrostatic repulsion, which inhibits adsorption [35]. Structurally, liposomes feature a classic phospholipid bilayer with an internal hydrophobic cavity [36]. Meanwhile, BSA also contains numerous hydrophobic amino acid residues. When exposed to certain environmental factors, the three-dimensional structure of BSA may partially unfold, causing its internal hydrophobic amino acids to become exposed on the molecular surface [37,38]. At this point, when BSA approaches the liposome, driven by hydrophobic interactions between the two, BSA may partially embed or fully insert into the liposome. Its hydrophobic regions become surrounded by the hydrophobic tails of the lipids, while its hydrophilic portions may remain exposed to the aqueous phase. This spontaneous insertion, driven by hydrophobic interactions, ultimately leads to the formation of the protein corona. In summary, the interaction between LP-0 and BSA likely follows an insertion model.

(ii) Adsorption model. When the cationic lipid DOTAP is introduced into the liposome composition, its positively charged quaternary ammonium head group imparts a significant positive charge to the entire liposome surface (Figure 1B). The positively charged liposomes and negatively charged BSA approach each other in solution due to Brownian motion. Once within the effective range of Coulombic forces, strong electrostatic interactions rapidly drive the adsorption of BSA onto the liposome surface [39]. This process primarily follows a surface adsorption model. This adsorption results in the formation of a protein corona around the liposome, whose binding mode and stability are primarily governed by the strength of the electrostatic interactions. Therefore, based on our hypothesis, both LP-3% and LP-6% likely form a surface adsorption model with BSA.

According to the surface adsorption model, liposomes adsorb large amounts of BSA onto their surface to form the protein corona. Consequently, the particle size should be larger and exhibit more significant changes compared to the insertion model, where BSA embeds into the liposomes. Additionally, the particle size is expected to increase with BSA concentration [40], which aligns with our experimental results (Figure 1E–J). Studies have shown that the formation of a protein corona can mask the original surface properties of nanoparticles, causing their biological identity to be defined by the outer layer of adsorbed proteins rather than the core material itself [41]. Changes in zeta potential confirmed these findings (Figure 2A,B). In the surface adsorption model, the abundant negatively charged amino acid residues distributed across the BSA surface can simultaneously and flexibly engage in electrostatic interactions with the multiple dispersed positively charged head groups of DOTAP on the liposome membrane surface [42]. This mode of contact allows for a large number of non-specific, low-energy-barrier binding sites in the liposome-BSA association. In contrast, for the insertion model, BSA must wedge its hydrophobic core or specific structural domains into the hydrophobic interior of the lipid bilayer. This process initially requires locally disrupting the orderly arrangement of lipid molecules and overcoming a high energy barrier. Consequently, the binding force required exceeds that of a purely electrostatic attraction. More importantly, this insertion behavior relies on the presence of specific sites on the BSA molecule that possess sufficient hydrophobicity and appropriate structure [43]. The number of such sites is considerably fewer than the widely distributed surface charge-based binding sites. Consequently, the total number of adsorption sites in the insertion model is lower than that in the simpler and more flexible surface adsorption model. The LP-0 exhibited lower BSA adsorption compared to LP-3% and LP-6% (Figure 2C). This observation was further supported by its substantially smaller change in zeta potential relative to LP-3% and LP-6% (Figure 2A,B).

Interestingly, a pronounced aggregation of LP-6% nanoparticles was observed following incubation, resulting in a significant increase in particle size. In contrast, no such aggregation was observed in any of the other groups (Figure 1C). To investigate the cause of this nanoparticle aggregation, photographs of the liposomes with or without BSA were taken after a 2 h incubation period, as shown in Figure 1D. The LP-6% group showed obvious precipitation after incubation compared to the original ones. Furthermore, changes in particle size and polydispersity index (PDI) for the three liposome formulations after incubation with varying BSA concentrations corresponded with the above findings (Figure 1E–J). This observation aligns with our initial hypothetical models. As discussed later in Figure 5, the conformational structure of BSA remains largely unchanged following incubation with liposomes. Therefore, the observed aggregation likely results from structural alterations in the liposomes induced by the addition of BSA. Research has shown that the stability of liposomes is significantly influenced by several physicochemical barriers, including electrostatic repulsion [44], steric hindrance [45], and membrane structural integrity [46]. As shown in Figure 2A, while the zeta potentials of both LP-6% and LP-3% approach zero, only LP-6% exhibited precipitation, ruling out the disruption of electrostatic repulsion as the cause. Based on literature findings, we hypothesize that the precipitation in LP-6% may be attributed to its higher surface positive charge density, which could lead to increased protein adsorption. Excessive protein adsorption may disrupt the steric hindrance between liposomes in the same environment, causing them to aggregate [47]. Additionally, the increased proportion of DOTAP in the liposomes may have compromised the lipid membrane structure, leading to the formation of unstable complexes that tend to form large clusters after incubation [48]. The measured protein adsorption also reflects this complex instability. Protein adsorption was determined by measuring free protein in the supernatant after ultracentrifugation. Unstable liposome–protein complexes dissociate under the high shear forces of ultracentrifugation, leading to an increased free protein measurement and a corresponding decrease in calculated adsorption. This explains why LP-6% shows lower adsorption than LP-3% (Figure 2C). In a related study, Sangrà et al. reported that liposomes composed of unsaturated phospholipids consistently aggregated in BSA and could not be effectively separated [49]. By comparison, our experimental results showed a different trend. Among the three liposomal formulations containing unsaturated phospholipids (egg yolk lecithin), only LP-6% exhibited aggregation under the same conditions, while LP-0 and LP-3% remained stable. This suggests that the aggregation behavior of liposomes in BSA is determined not solely by the presence of unsaturated phospholipids, but more critically by their proportion within the liposomal formulation. Our findings further indicate that by precisely adjusting the percentage of unsaturated phospholipids, one can actively modulate both protein corona formation and the colloidal stability of liposomes. These insights provide important guidance for the rational design of liposomal systems. This study simulated the aggregation that may occur after nanomedicines enter the body, indicating potential in vivo risks [50].

Meanwhile, we observed that the presence of MgCl_2_ also appears to influence the interaction between BSA and the liposomes. Upon the addition of MgCl_2_, the detected zeta potential shifted closer to that of the original liposomes (Figure 2B). Additionally, the amount of adsorption decreased across all groups (Figure 2C). This intriguing phenomenon leads us to consider the role played by MgCl_2_ in the liposome-BSA system. Studies indicated that metal ions can interact with proteins through both specific and non-specific binding [51,52]. Meanwhile, they could also bind to the headgroups of negatively charged liposomes via electrostatic interactions [53]. When Mg^2+^ is introduced into a mixed system of proteins and liposomes, it acts as both a competitive binder and a shielding agent. By simultaneously occupying potential binding sites on both the protein and liposome surfaces, they create a barrier that hinders direct interaction between the two. Therefore, the addition of MgCl_2_ likely reduces the interaction between them, leading to decreased BSA adsorption on the liposomes and diminishing the masking effect of the adsorbed BSA on the liposomal surface properties. This interpretation aligns with our experimental results. In their study, Huang et al. demonstrated that the presence of inorganic ions can enhance the interaction forces between liposomes and proteins, thereby promoting protein adsorption on the liposome surface and facilitating the formation of a protein corona [30]. In contrast to those findings, our results revealed the complex regulatory role of ions present in physiological fluids on liposome–protein interactions. The findings indicate that ions may not only promote protein adsorption through mechanisms such as chelation or electrostatic screening, but could also impede interactions between proteins and the liposomal surface via competitive binding, steric hindrance, or induced conformational changes. This dual regulatory capacity underscores the more intricate and dynamic role ions play at biological interfaces. Therefore, experimental investigations must comprehensively consider the physicochemical properties of the specific system and the environmental conditions involved.

**Figure 3:**
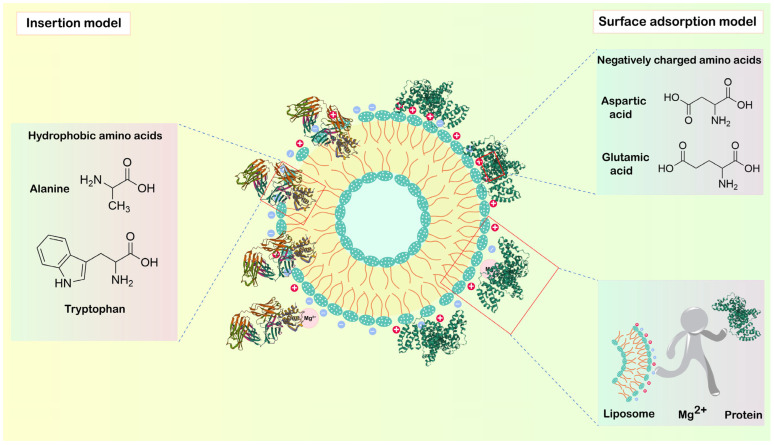
The hypothetical models of protein corona formation.

In summary, based on the proportion of DOTAP in the liposome composition, we introduced the (i) insertion model and (ii) surface adsorption model. Furthermore, we presented evidence that MgCl_2_, an inorganic salt present in physiological environments, reduced the interaction between liposomes and BSA. Schematic diagrams of these hypothetical models are shown in Figure 3.

### 3.3. Validation of Protein Corona Formation Models

**Figure 4:**
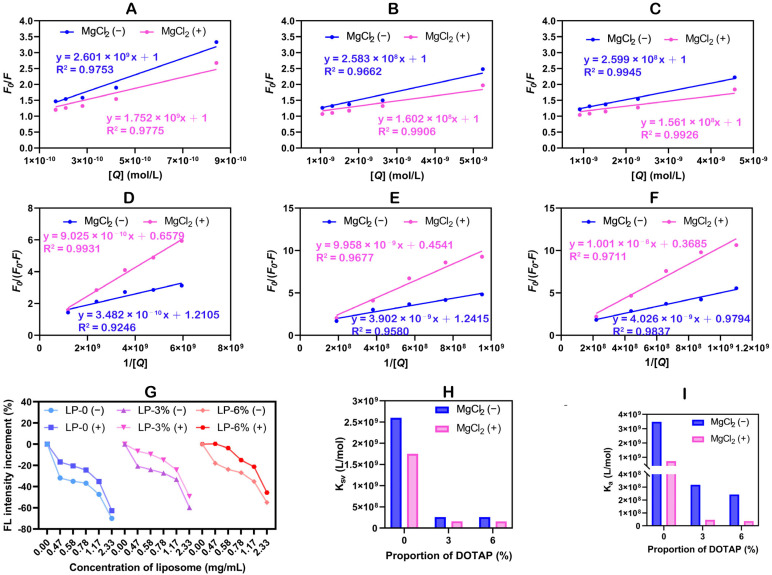
The (**A**–**C**) Stern–Volmer plots and (**D**–**F**) the modified Stern–Volmer plots of BSA incubated with liposomes. (**G**) The fluorescence intensity increment of BSA incubated with liposomes at different concentrations. The *K*_sv_ values (**H**) and the *K*_a_ values (**I**) of BSA incubated with liposomes.

To further validate the above model, we analyzed the binding mode between BSA and liposomes by measuring fluorescence quenching spectra. When excited at 280 nm, BSA exhibits strong and stable intrinsic fluorescence due to its chromophore amino acids (AAs), such as tryptophan (Trp), tyrosine (Tyr), and phenylalanine (Phe) [54]. We measured the fluorescence quenching of BSA in the presence of different concentrations of liposomes. As the liposome concentration increased, the fluorescence emission intensity of BSA gradually decreased, meaning the difference compared to the control group (BSA alone) became larger. This indicated that there was an interaction between BSA and the liposomes (Figure 4G). To further clarify the mechanism behind the fluorescence quenching induced by liposomes, we analyzed the quenching data using the Stern–Volmer equation, as shown in Equation (1) [55]:

(1) $$\frac{F_{0}}{F}=1+K_{sv}\left[Q\right]=1+K_{q}\tau_{0}[Q]$$

In the equation, *K*_sv_ is the quenching constant, which is used to indicate the strength of the interaction between substances. As shown in Figure 4A–C, the fluorescence quenching plots of BSA with liposomes at various concentrations exhibit strong linear fitting, indicating the presence of either a single dynamic quenching mechanism or a static quenching mechanism. The *K*_sv_ values were calculated from the slopes of the equations (Figure 4H). The results showed that the *K*_sv_ of LP-0 is significantly greater than that of LP-3% and LP-6%, indicating that the interaction between BSA and liposomes in the insertion model is stronger than that in the surface adsorption model, resulting in a greater degree of quenching. Furthermore, the addition of MgCl_2_ resulted in a decrease in the *K*_sv_ of the liposomes, meaning a lower quenching efficiency. This indicates that MgCl_2_ weakens the interaction between BSA and the liposomes. The incorporation of Mg^2+^ leads to a looser binding between the liposomes and BSA, resulting in a naturally weaker interaction compared to the stronger electrostatic forces in the model. The data in Figure 4G also support this conclusion. Additionally, the binding affinity constant (*K*_a_) between BSA and the liposomes can be calculated using the modified Stern–Volmer equation. This constant reveals the strength of their interaction and the stability of the complex formed. The modified Stern–Volmer equation is expressed as Equation (2) [56]:

(2) $$\frac{F_{0}}{F_{0}-F}=\;\frac{1}{f_{a}}+\frac{1}{f_{a}K_{a}[Q]}$$

Here, *f*_a_ represents the fraction of accessible fluorescence. Similarly, the *K*_a_ value was calculated using the equation (Figure 4I), and the results show that the variation trend of *K*_a_ is consistent with that of the *K*_sv_ values. In summary, the fluorescence spectroscopy data are in full agreement with our proposed model.

### 3.4. Protein Conformational Studies

**Figure 5:**
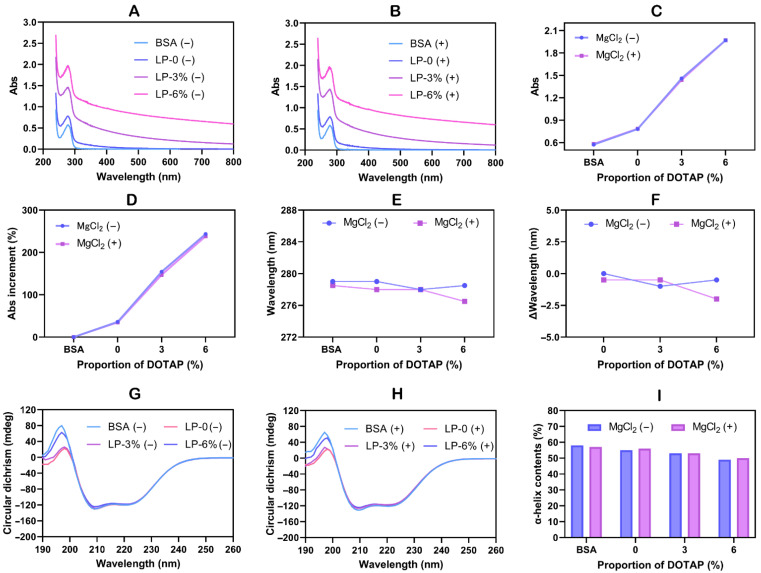
The UV-vis spectra of BSA incubated with liposomes in (**A**) without MgCl_2_ (−) and (**B**) with MgCl_2_ (+). The absorbance peak for BSA was recorded at a wavelength of 279 nm (without MgCl_2_) and 278 nm (with MgCl_2_). The (**C**) absorbance and the (**D**) ∆absorbance at 279 nm or 278 nm of BSA incubated with liposomes. The (**E**) wavelength and the (**F**) ∆wavelength of the characteristic absorption peak of BSA incubated with liposomes. The CD spectra of BSA incubated with liposomes in (**G**) without MgCl_2_ (−) and (**H**) with MgCl_2_ (+). (**I**) The α-helix structure percentage of BSA incubated with liposomes.

To further investigate the interaction process between liposomes and BSA, changes in the protein’s conformation were evaluated using UV-vis and CD spectroscopy. The UV-vis results showed that the absorbance of BSA at 279 nm (without MgCl_2_) (Figure 5A) and 278 nm (with MgCl_2_) (Figure 5B) increased significantly after incubation, indicating greater exposure of chromophore AAs. However, the extent of absorbance change was similar in systems with and without MgCl_2_ (Figure 5C,D), and no differences were observed in the absorption wavelengths or their shifts (Figure 5E,F). This suggests that neither the adsorbed liposomes nor the presence of MgCl_2_ in the system alters the structure of BSA. To further verify our hypothesis, we measured the CD spectrum of BSA after incubation with liposomes, as shown in Figure 5G–H. It is known that the CD spectrum of BSA displays negative peaks at 222 nm and 208 nm, which are characteristic signals of its α-helical structure [57]. The specific percentage of α-helical structure was summarized using the CDSSTR algorithm, as shown in Figure 5I. The results are consistent with the findings from the UV-vis spectroscopy. In summary, the addition of either DOTAP or MgCl_2_ only altered the mode of interaction between the liposomes and BSA, without disrupting the structure of the protein itself. This indicates that the interaction between liposomes and proteins is relatively weak and proceeds through a mild process, which holds significant implications. It demonstrates that liposomes can serve as excellent protein carriers, capable of preserving the activity of biopharmaceuticals to the greatest extent in drug delivery applications.

### 3.5. Cellular Uptake

**Figure 6:**
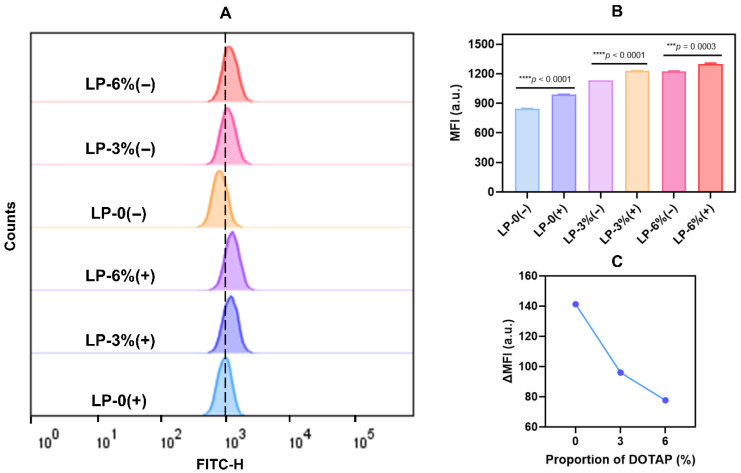
(**A**) Analysis of nanoparticle uptake by RAW264.7 cells under different treatments using FCM, and (**B**,**C**) quantitative analysis of the uptake efficiency. The symbol (−) indicates the absence of FBS in the system, while (+) represents the presence of FBS.

After confirming the validity of our model, we proceeded to investigate the biological effects resulting from the interaction between liposomes and cells following the formation of a protein corona. Cellular uptake is an essential pathway for the internalization of nanoparticles, and RAW264.7 cells serve functions in immune surveillance, which were utilized as the cellular model to explore the impacts on cellular uptake. We incubated liposomes, both with and without 10% fetal bovine serum (FBS), with RAW264.7 cells under identical culture conditions. The uptake efficiency of the protein corona-coated liposomes by RAW264.7 cells was then characterized using flow cytometry (FCM) (Figure 6A–C). The results showed that as the proportion of DOTAP increased, resulting in a more positive zeta potential, cellular uptake of the nanoparticles was enhanced. This observation aligns with our proposed model. In the surface adsorption model, the relatively weak interaction between liposomes and BSA results in a limited shielding effect, allowing the intrinsic properties of the inner liposome to remain exposed. Consequently, the strong electrostatic attraction between the negatively charged cell membrane and the positively charged liposome enhances cellular uptake of the nanoparticles. Consistent with this phenomenon, the study by Ibuki et al. also demonstrated that an increase in zeta potential significantly enhanced the uptake efficiency of liposomes by macrophages [58]. This further elucidated the intrinsic relationship between the chemical composition of liposomes, the formation of the protein corona, and cellular uptake, thereby providing a mechanistic theoretical basis for explaining the behavioral differences in various liposomes in biological systems. Furthermore, uptake was stronger in the groups containing FBS compared to those without FBS. This phenomenon can be explained from the perspective of complement proteins. FBS is a rich source of complement proteins, and components such as C1q and C3b act as opsonins. When these complement proteins adsorb onto the surface of liposomes, they can specifically bind to complement receptors (e.g., CR1, CR3) on RAW264.7 cells, thereby significantly enhancing phagocytic recognition and internalization of the targeted particles [59,60]. Consequently, the introduction of FBS leads to a stronger uptake phenomenon by activating the complement-dependent opsonophagocytic pathway. The study holds significant implications for fields such as cancer immunotherapy [61] and the integration of chemotherapy with gene therapy [62]. It provided experimental evidence and preliminary mechanistic insights for precisely regulating the in vivo fate of nanomedicines. However, further investigation and more extensive biological validation are still required in future research.

In conclusion, this study provided both key theoretical and experimental foundations for precisely controlling the in vivo fate of nanomedicines, establishing a theoretical basis for achieving highly efficient targeted delivery.

## 4. Conclusions

In this study, we investigated how surface charge and the inorganic salt MgCl_2_ influence the interaction between liposomes and BSA, aiming to elucidate different patterns of protein corona formation. By analyzing changes in particle size, zeta potential, and adsorption amount after incubating BSA with liposomes, we observed that the addition of both DOTAP and MgCl_2_ alters the interaction mode between BSA and the liposomes. Based on these findings, we have proposed two distinct hypothetical models. Based on the proportion of DOTAP, we proposed an insertion model and a surface adsorption model. LP-0 corresponds to the insertion model, while LP-3% and LP-6% follow the surface adsorption model. In the insertion model, the interaction strength is greater than that in the surface adsorption model, but the number of binding sites is fewer, resulting in a lower overall adsorption amount. The addition of MgCl_2_ hindered direct binding between the liposomes and BSA, leading to a significant reduction in their interaction strength. Subsequent spectroscopic analysis validated the rationality of the hypothetical models constructed above. The results showed that the presence of both DOTAP and MgCl_2_ in the system led to a decrease in the *K_SV_* and *K_a_* values, indicating that the addition of either component weakens the interaction forces between the liposomes and proteins. We believe these theoretical models help reveal key interactions and behavioral patterns of liposomes in complex biological environments, providing new insights for the design and application of nanocarriers in the field of drug delivery.

## Data Availability

The original contributions presented in this study are included in the article and Supplementary Materials. Further inquiries can be directed to the corresponding authors.

## Supplementary Materials

The following supporting information can be downloaded at: https://www.mdpi.com/article/10.3390/pharmaceutics18010076/s1, Table S1: Study comparison.
