# Supplementary material for: Charge Effects: Influence of Surface Charge on Protein Corona Adsorption Behavior on Liposomal Formulations
Source: Pharmaceutics. 2026 Jan 7;18(1):76. doi: 10.3390/pharmaceutics18010076 (PMC12844955; doi:10.3390/pharmaceutics18010076)
Supplement: Supplementary file 1 [file pharmaceutics-18-00076-s001.zip › pharmaceutics-4021033-supplementary.pdf]

Charge Effects: Influence of Surface Charge on Protein Corona Adsorption Behavior on Liposomal Formulations

Qian Chen [1,†], Yeqi Huang [1,†], Chuanbin Wu [2], Xin Pan [3], Changjiang Yu [4], Jiu Wang [5], Wenhao Wang [3,]* and Zhengwei Huang [2,6,]*

Table S1 Study comparison

Study	Advantages	Limitations	Yield	Versatility
This work	Examines dual regulation by charge (DOTAP) and ionic environment (Mg^{2+}); Introduces Mg^{2+} to simulate physiological ionic conditions; Offers new insights for rational liposome design in complex biological fluids.	Possibly limited to a single-protein (BSA) model, differing from real plasma corona complexity; Lacks systematic investigation of dose-dependent effects of DOTAP ratio and Mg^{2+} concentration; Lacks *in vivo* or more complex *in vitro* model validation.	Lacks *in vivo* or more complex *in vitro* model validation; Provides experimental basis for modulating protein corona by tuning charge and ionic environment.	Provides a reference for designing smart liposomes adapted to specific physiological environments (e.g., tumor microenvironment, inflammatory sites); Potentially extendable to studies with other divalent ions (e.g., Ca^{2+}) or mixed protein systems.
[12] DOI: 10.1021/acsomega.3c09131	Systematically compares CHOL vs. DOTAP effects on BSA adsorption; Clearly shows DOTAP promotes protein adsorption but induces aggregation, while CHOL enhances stability.	Uses only BSA, not complex serum protein environments; Does not investigate the effects of ion type or concentration on adsorption behavior.	Quantifies differences in BSA adsorption between CHOL and DOTAP liposomes; shows DOTAP induces aggregation; Highlights limitations and applicability of separation methods (centrifugation vs. SEC).	Offers fundamental data for charge-based liposome design (e.g., optimization of gene delivery vectors); Expandable to studies with other charged lipids or serum protein adsorption.
[58] DOI: 10.1248/bpb.b23-00755	Clarifies the membrane fluidity - protein adsorption - cellular uptake mechanism chain; Provides rationale for designing liposomes targeting macrophages.	Does not explore the impact of surface charge variation (e.g., using other charged lipids) on protein corona; Does not systematically study other ions or environmental factors.	Establishes a clear link: Chol ratio - membrane fluidity - protein adsorption - cellular uptake efficiency; Identifies key roles of specific adsorbed proteins (e.g., apolipoproteins) in mediating uptake.	Guides formulation optimization for macrophage-targeted drug delivery systems (e.g., anti-infective, immunotherapy); Expandable to validation in other cell types or animal models.
